# The association between oral and gut microbiota in male patients with alcohol dependence

**DOI:** 10.3389/fmicb.2023.1203678

**Published:** 2023-07-28

**Authors:** Lingming Hu, Zhaojun Ni, Kangqing Zhao, Xiangxue Li, Xuejiao Gao, Yulin Kang, Zhoulong Yu, Ying Qin, Jingwen Zhao, Wenjuan Peng, Lin Lu, Hongqiang Sun

**Affiliations:** ^1^Peking University Sixth Hospital, Peking University Institute of Mental Health, NHC Key Laboratory of Mental Health (Peking University), National Clinical Research Center for Mental Disorders (Peking University Sixth Hospital), Peking University, Beijing, China; ^2^Chinese Research Academy of Environmental Sciences, Beijing, China; ^3^The Second People’s Hospital of Guizhou Province, Guiyang, Guizhou, China; ^4^National Institute on Drug Dependence, Beijing Key Laboratory of Drug Dependence, Peking University, Beijing, China; ^5^Peking-Tsinghua Centre for Life Sciences and PKU-DG/McGovern Institute for Brain Research, Peking University, Beijing, China

**Keywords:** alcohol dependence, oral microbiota, gut microbiota, ectopic colonization, Source Tracker

## Abstract

**Introduction:**

The relationship between oral and gut microbiota in alcohol dependence (AD) is not well understood, particularly the effects of oral microbiota on the intestinal microbiota. The current study aimed to explore the association between oral and gut microbiota in AD to clarify whether oral microbiota could ectopically colonize into the gut.

**Methods:**

16S rRNA sequence libraries were used to compare oral and gut microbial profiles in persons with AD and healthy controls (HC). Source Tracker and NetShift were used to identify bacteria responsible for ectopic colonization and indicate the driver function of ectopic colonization bacteria.

**Results:**

The α-diversity of oral microbiota and intestinal microbiota was significantly decreased in persons with AD (all *p* < 0.05). Principal coordinate analysis indicated greater similarity between oral and gut microbiota in persons with AD than that in HC, and oral-gut overlaps in microbiota were found for 9 genera in persons with AD relative to only 3 genera in HC. The contribution ratio of oral microbiota to intestinal microbiota composition in AD is 5.26% based on Source Tracker，and the AD with ectopic colonization showed the daily maximum standard drinks, red blood cell counts, hemoglobin content, and PACS scores decreasing (all *p* < 0.05).

**Discussion:**

Results highlight the connection between oral-gut microbiota in AD and suggest novel potential mechanistic possibilities.

## Introduction

Alcohol dependence (AD) causes physical and psychological damage to patients and contributed to more than 3 million deaths in 2016, accounting for 5.3 percent of total global mortality (World Health Organization, 2018). Alcohol dependence was the most prevalent substance use disorder in China with a lifetime prevalence of 1.3% and a 12-month prevalence of 0.7% as of 2015 (Huang et al., 2019). The pathogenesis of AD remains unclear, although recent studies have implicated dysfunction of the oral or gut microbiota correlated with alcohol dependence (Leclercq et al., 2014; Dubinkina et al., 2017). Previous study suggested that oral and gut microbiota as well as their products may have an impact on nutritional metabolism, immune protection, and neuro-regulation in individuals from infancy to senescence (Hehemann et al., 2010; Adak and Khan, 2019). Meanwhile, gut microbiota has a profound influence on the progression of AD and animal studies have shown that fecal microbiota transplantation from AD into germ-free mice interferes with recipient cognitive function, induces anxiety/depression behavior decreases expression of brain-derived neurotrophic factor (BDNF), and the function of mGluR1/PKC ε pathway in the brain (Zhao et al., 2020). In addition, oral bacteria, such as *Neisseria*, are known to participate in the metabolism of nitrate and acetaldehyde, perhaps contributing to AD-related cognitive impairment (Fan et al., 2018; Rosier et al., 2020). And increased levels of *P. gingivalis*, resulting from a disturbed balance of the oral microbiome, may impair the function of the blood–brain barrier (BBB) and elicit neuroinflammation (Panza et al., 2019).

Both oral and gut microflora may thus contribute to the pathogenesis of AD, but little is known about the association between the two bacterial populations. Healthy people generate and swallow about 1.5 L of saliva per day containing an enormous number of oral microbiota and metabolites (Humphrey and Williamson, 2001; Atarashi et al., 2017). Normal intestinal barrier function would prevent cross-colonization of oral and gut microbiota in healthy individuals, but the loss of intestinal integrity has been shown to cause invasion of oral bacteria into the intestine, like gastrointestinal diseases or in germ-free animal models (Humphrey and Williamson, 2001; Bull-Otterson et al., 2013; Atarashi et al., 2017; Park et al., 2021). The phenomenon of oral bacteria found in the gut is one type of ectopic colonization in bacteria that has been observed in many disease states already (Strandwitz, 2018; Rashidi et al., 2021). More oral microbiota has been found colonized in the gut of irritable bowel syndrome (IBS), inflammatory bowel disease (IBD), and colorectal cancer (CRC), compared with healthy individuals (Gevers et al., 2014; Iwauchi et al., 2019; Pittayanon et al., 2019; Thomas et al., 2019). Such ectopic colonization might aggravate the disturbed gut microbiota and immunoreactivity. Effects of oral microbiota on gut microbiota have been found to contribute to microglia-mediated neuroinflammation in major depression disorder (MDD) (Scassellati et al., 2021), and heavy alcohol consumption is known to impair gut barrier function and cause dysfunction of oral and gut microbiota (Forsyth et al., 2009; Leclercq et al., 2017). Therefore, the association between oral and gut microbiota seems to affect the progression of AD, but this association is still unclear.

The current study investigated whether oral microbiota could be ectopically colonized into the gut and whether those ectopic colonization bacteria are related to clinical features in AD. Findings from this study may contribute to a better understanding of oral-gut microbiota in AD, expose potential mechanisms and inform therapeutic strategies of AD in clinical practice.

## Materials and methods

### Participants

Thirty-three male patients diagnosed with alcohol dependence were recruited from the Second People’s Hospital of Guizhou Province during July and September 2019. alcohol dependence was diagnosed using the Diagnostic and Statistical Manual of Mental Disorders, 4th edition (DSM-IV). Other than nicotine dependence, as determined by the Mini International Neuropsychiatric Interview (M.I.N.I), patients did not present with other substance use disorders or other DSM-VI mental health or Axis I disorders. Twenty-one male healthy controls (HC) without somatic and psychiatric illnesses were also recruited. HC did not have a current or lifetime history of alcohol use or other substance use disorders. The inclusion and exclusion criteria are shown below.

The inclusion and exclusion criteria are shown below. The inclusion criteria for the alcohol dependence (AD) group were as follows: (1) diagnosis of AD according to the Diagnostic and Statistical Manual of Mental Disorders, 4th edition (DSM-IV); (2) Han Chinese males aged 18–60 years. The exclusion criteria for the AD group were as follows: (1) had or having the infectious disease; (2) had or having heart, brain, liver, kidney, oral and other serious diseases; (3) had or having metabolic diseases that can lead to the abnormal immune system; (4) had or having neurodegenerative diseases; (5) used antibiotics, steroids or other microbiota-modulating medications within 2 months before enrollment; (6) previous or current DSM-IV diagnosis of schizophrenia, depression, anxiety disorder, bipolar disorder, mental retardation, dementia (excluding mild cognitive function), and substance dependence other than alcohol and nicotine; (7) had irregular eating habits that affected oral flora (except alcohol) in recent 2 months; (8) alcohol abstinence longer than 1 week. The inclusion criteria for the HC group were as follows: Han Chinese males aged 18–60 years. The exclusion criteria for the HC group were as follows: (1)–(8) exclusion criteria for patients with alcohol dependence but (6) patients with a previous or current DSM-IV diagnosis of schizophrenia, depression, anxiety disorder, bipolar disorder, mental retardation, dementia (excluding MCI), and substance dependence, including alcohol (excluding nicotine); (9) drinking alcohol during sample collection.

G*Power3.1.9.7 software was used to calculate the sample size, and it was assumed that the test standard α = 0.05 (bilateral), the effect value was 0.5, the beta value was 0.20, and the degree of certainty (1-β) was 0.80. It was calculated that the minimum sample size required was 18.

All subjects gave written informed consent for the collection of saliva and feces and participation in the study.

All experiments were conducted by the Helsinki Declaration of 1975 and the ethical standards of the home institution Committee on Human Experimentation. Ethical approval was granted by the ethics committees of Peking University Sixth Hospital (2019, ethical review No. 6) and the Second People’s Hospital of Guizhou Province.

### Clinical features and assessment scales

Clinical features of AD participants were indexed, including withdrawal days, dependence years, first drinking age (FD age), frequency of drinking weekly (FREW), frequency of drinking daily (FRED), average standard drinks per day (ASD), maximum standard drinks per day (MSD), smoking status, body mass index (BMI), aspartate aminotransferase (AST), alanine transaminase (ALT), gamma-glutamyl transpeptidase (GGT), total bilirubine (TBIL), red blood cell (RBC), hemoglobin (HB), mean corpuscular volume (MCV), triiodothyronine (TT3), tetraiodothyronine (TT4), thyroid stimulating hormone (TSH); alcohol withdrawal syndrome (evaluated by Clinical Institute Withdrawal Assessment for Alcohol-Revised, CIWA-Ar), alcohol craving (evaluated by Pennsylvania Alcohol Craving Scale, PACS), cognitive function (evaluated by Montreal Cognitive Assessment, MoCA), nicotine consumption (evaluated by Fagerstrom test for nicotine dependence, FTND), sleeping patterns (evaluated by Pittsburg Sleep Quality Index, PSQI) and symptoms of anxiety /depression (evaluated by Hamilton Anxiety Scale and Hamilton Depression scale, HAMA and HAMD) were recorded. Control subjects were assessed for cognitive function, depression and anxiety, and sleeping patterns.

### Sample collection

All subjects fasted overnight and did not brush their teeth. Fresh saliva and feces were collected at 7:00 to 8:00 am into sterile EP tubes. Saliva was collected after patients had rinsed the mouth with water 5 min beforehand and 1–2 ml of naturally secreted saliva was removed by Pap dropper into an EP tube. The procedure was repeated once. Feces were collected after patients had urinated to avoid contamination and defecated into a clean bedpan, from which 1–2 g stool, which had not been exposed to air, was removed with a sampling spoon. The procedure was repeated once. All samples were frozen in liquid nitrogen for 1 min and stored at −80°C. All samples were collected by procedures specified in the Human Microbiome Project (HMP) version 12 standard process (Integrative HMP Research Network Consortium, 2019).

### DNA extraction

DNA was extracted from the oral and gut microbial community using NEBnext microbiome DNA enrichment kit (New England Biolabs, Ipswich, MA, US). The detailed procedure following the manufacturer’s instructions, including 600 μl Buffer with magnetic beads +20 μl Proteinase K + 5 μl RNase A was added to 96-well deep plates before washing three times and adding 100 μl Elution Buffer. 100–200 mg sample was transferred to the centrifuge tube with grinding beads and 1 ml Buffer (ATL/PVP-10) added and the sample was ground and incubated at 65°C for 20 min before centrifugation at 14000 × g for 5 min. 0.6 ml Buffer PCI was added with mixing by vortex for 15 s and centrifugation at 12000 × *g* for 10 min. The supernatant was transferred to a deep well plate with magnetic beads binding solution and DNA was transferred to a 1.5 ml centrifuge tube. DNA concentration and purity were assessed by NanoDrop 2000 micro-ultraviolet spectrophotometer and quantified with a Qubit Fluorometer using Qubit® dsDNA BR Assay kit (Invitrogen, USA). DNA quality was checked by running an aliquot on 1% agarose gel.

### 16S rRNA sequencing and analysis

Variable regions, V3 ~ V4, of the bacterial 16S rRNA gene, were amplified with degenerate PCR primers, 515F (5′-GTGCCAGCMGCCGCGGTAA-3′) and 806R (5′-GGAC TACHVGGGTWT-CTAAT-3′) and products purified by Agencourt AMPure XP beads and eluted with elution buffer. Agilent Technologies 2,100 bioanalyzer qualified the Libraries and sequenced using an Illumina HiSeq 2,500 platform (BGI, Shenzhen, China), following the manufacturer’s instructions. 2 × 250 bp paired-end reads were generated. Raw reads were filtered and paired-end reads were tagged for clustering into operational taxonomic units (OTUs). OTU representative sequences were taxonomically classified using Ribosomal Database Project (RDP) Classifier v.2.2 with a minimum confidence threshold of 0.6 and trained on the Green-genes database v201305 by QIIME 2 (Bolyen et al., 2019). Alpha and beta diversity were estimated by MOTHUR (v1.31.2) and QIIME 2, respectively (Schloss et al., 2009; Wade and Prosdocimi, 2020).

Different classification levels were plotted with R package v3.4.1 and R package “gg plots” (Shamsheer et al., 2022). Principal coordinate analysis (PCoA) of OTUs was conducted by R package “ade4” (Jombart, 2008). Wilcoxon or Kruskal–Wallis’ testing was used to ascertain significant species or functions by R (v3.4.1) software.

DNA extraction, 16S rRNA sequencing, and analyses were performed by BGI Co., LTD. Shenzhen, China.

### Source Tracker and NetShift analyses

Source Tracker is a Bayesian approach to allow the estimation of the proportion of contaminants in a given community that come from possible source environments (Knights et al., 2011). Oral microbiota was considered source environments and gut microbiota sink environments for calculation of the contribution rate of oral microbiota to gut microbiota. Source Tracker R script was downloaded from https://github.com/danknights/sourcetracker. NetShift was used to quantify oral and gut community changes between the AD and HC to identify the microbial taxa which serve as “driver-bacteria,” promoting the change from the healthy to the diseased state. The correlation network was established based on the Pearson coefficients between bacteria communities in AD and HC. Each genus was regarded as a point in the network and the genus with a significant correlation relationship was assigned an edge (Kuntal et al., 2019). The Netshift application was used to elucidate the drivers of HC to the AD network. NetShift algorithm could be downloaded from NetShift was downloaded from https://web.niapps.net/netshift.

### Statistical analysis

Statistical analyses were performed with IBM SPSS 23.0, R-4.2.0, and Graphpad prism 8.0.2. Differences in numerical variables between AD and HC, continuous variables consistent with normal distribution and homogeneity of variance were assessed by a two-tailed Student’s *t*-test and results expressed as the mean ± standard deviation, other continuous variables assessed by nonparametric test and results expressed as the median (Q25, Q75). Discontinuous variables were analyzed by the chi-square test. The *α*-diversity index and *β*-diversity index were calculated and compared between HC and AD microbiota. Principal coordinate analysis (PCoA) was used to evaluate the species differences in the microbiota between different sample groups. Based on the Wilcoxon test, calculated the characteristics of composition in oral/gut microbiota between AD and HC from phylum to genus. Oral-gut overlap microbiota based on common OTUs number by VeenDiagram package in R. Comparison of clinical features between ectopic colonization (EC) patients and no ectopic colonization (NE) patients by Mann–Whitney U test. Correlational analyses were assessed by the Spearman test. The value of *p* < 0.05 was considered statistically significant.

## Results

### Baseline characteristics of alcohol dependence and healthy controls

No significant differences were present in age, occupation, educational status, marital status, annual income, BMI, or smoking status. No significant difference in FTND was found, but HAMD, HAMA, and PSQI scores were higher for AD than for HC (*p* < 0.001), and MoCA scores were lower for AD than for HC (*p* < 0.05). Demographic and clinical characteristics of the study population are summarized in Table 1.

**Table 1:** Baseline characteristics in alcohol dependence and healthy controls.

		AD *n* = 33	HC *n* = 21	*χ*^2^/t/z	Value of *p*
Age, year		44.00 (36.50, 51.00)	43.00 (27.00, 51.00)	−1.05	0.30
Occupation	In-service	24	15	0.01	0.92
	Unemployed	9	6		
Education years		7.36 ± 4.26	8.57 ± 4.38	−1.01	0.32
Marriage	Married	28	15	1.43	0.23
	Not married	5	6		
Smoking status	Yes	30	16	3.19	0.07
	No	3	6		
Income		60000.00 (15000.00, 100000.00)	30000.00 (10000.00, 55000.00)	−1.60	0.11
BMI, kg/m^2^		21.60 ± 2.29	22.87 ± 3.08	−1.73	0.09
MoCA		19.60 ± 6.41	23.79 ± 5.41	−2.39	0.02
FTND		4.00 (2.00, 6.00)	4.00 (0.00, 6.00)	−0.69	0.49
HAMA		7.00 (2.00, 10.50)	1.00 (0.25, 2.75)	−4.32	<0.001
HAMD		7.00 (1.50, 11.00)	1.00 (0.00, 2.00)	−3.71	<0.001
PSQI		7.00 (4.00, 15.50)	2.50 (1.00, 4.00)	−3.76	<0.001
Withdrawal days		2.00 (1.00, 4.00)	NA	NA	NA
AD years		10.00 (5.00, 17.00)	NA	NA	NA
FD age		16.00 (15.00, 18.00)	NA	NA	NA
ASD		15.00 (7.50，23.00)	NA	NA	NA
MSD		27.00 (13.00, 45.00)	NA	NA	NA
CIWA-Ar		17.33 ± 7.94	NA	NA	NA
PACS		5.00 (2.50, 9.00)	NA	NA	NA

AD, alcohol dependence; HC, healthy controls; BMI, Body Mass Index; MoCA, Montreal Cognitive Assessment Scale; FTND, Fagerstrom Test for Nicotine Dependence; HAMA, Hamilton Anxiety Scale; HAMD, Hamilton Depression Scale; PSQI, Pittsburg Sleep Quality Index; FD age, First Drinking age; ASD, Average Standard Drinks per day; MSD, Maximum Standard Drinks per day; CIWA-Ar, Clinical Institute Withdrawal Assessment for Alcohol-Revised; PACS, Pennsylvania Alcohol Scale.

### Oral and gut microbiota composition in alcohol dependence and healthy controls

**Figure 1:**
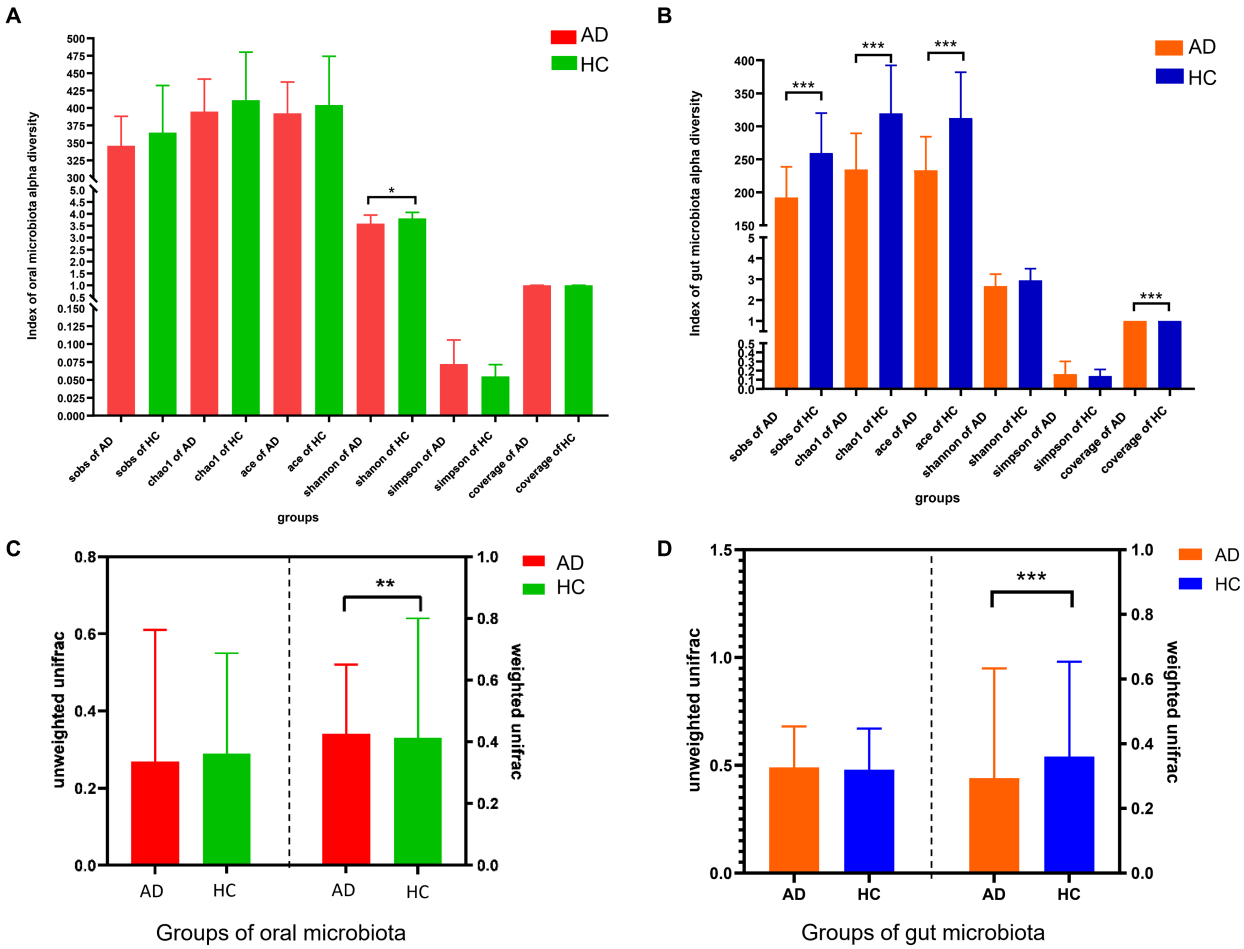
Differences in alpha and beta diversity between alcohol dependence and healthy controls. **(A)** α-diversity analysis showed that compared with the HC, the Shannon index of oral flora in the AD was lower, *p* < 0.05; no statistical difference was seen between AD and HC in the Sobs, Chao 1, ACE, Simpson, and Good coverage indices. **(B)** α-diversity analysis showed that compared with the HC, the intestinal flora observed species, Chao1, ACE and Good Coverage indices of the AD were lower, all *p* < 0.001; no statistical difference between AD and HC in the Shannon, Simpson indices. **(C)** Unweighted similarity analysis of β-diversity analysis showed that there was no statistical difference in β-diversity between the AD and HC, when sample sequence abundance was not considered; the results of weighted similarity analysis showed that there was a statistical difference in β-diversity between AD and HC, *p* < 0.01, when sample sequence abundance was considered. **(D)** Unweighted similarity analysis showed no statistical difference in β-diversity between AD and HC, when the sample sequence abundance was not considered; the results of the weighted similarity analysis showed a statistical difference in β-diversity between AD and HC, *p* < 0.001, when the sample sequence abundance was considered. All *p*-values are Bonferroni adjusted. * Indicates *p* < 0.05, ** indicates *p* < 0.01, *** indicates *p* < 0.001. AD indicates alcohol dependence, HC indicates healthy controls.

The *α*-diversity of oral microbiota (Shannon index) and intestinal microbiota (Sob index, Chao1 index, ACE index, and Coverage index) was significantly decreased in AD, compared with HC in Figures 1A,B (all *p* < 0.05). Based on weighted Unifrac, the *β*-diversity of oral microbiota between AD and HC exhibited statistical difference (*p* < 0.01); and based on weighted Unifrac, the *β*-diversity of gut microbiota between AD and HC exhibited statistical difference (*p* < 0.001) in Figures 1C,D. Compared with HC, the characteristics of composition in the oral and gut microbiota of AD from phylum to genus were summarized based on the Wilcoxon test (all *p* < 0.05), in Supplementary Figures S1, S2.

### Comparison of oral and gut microbiota in alcohol dependence and healthy controls

**Figure 2:**
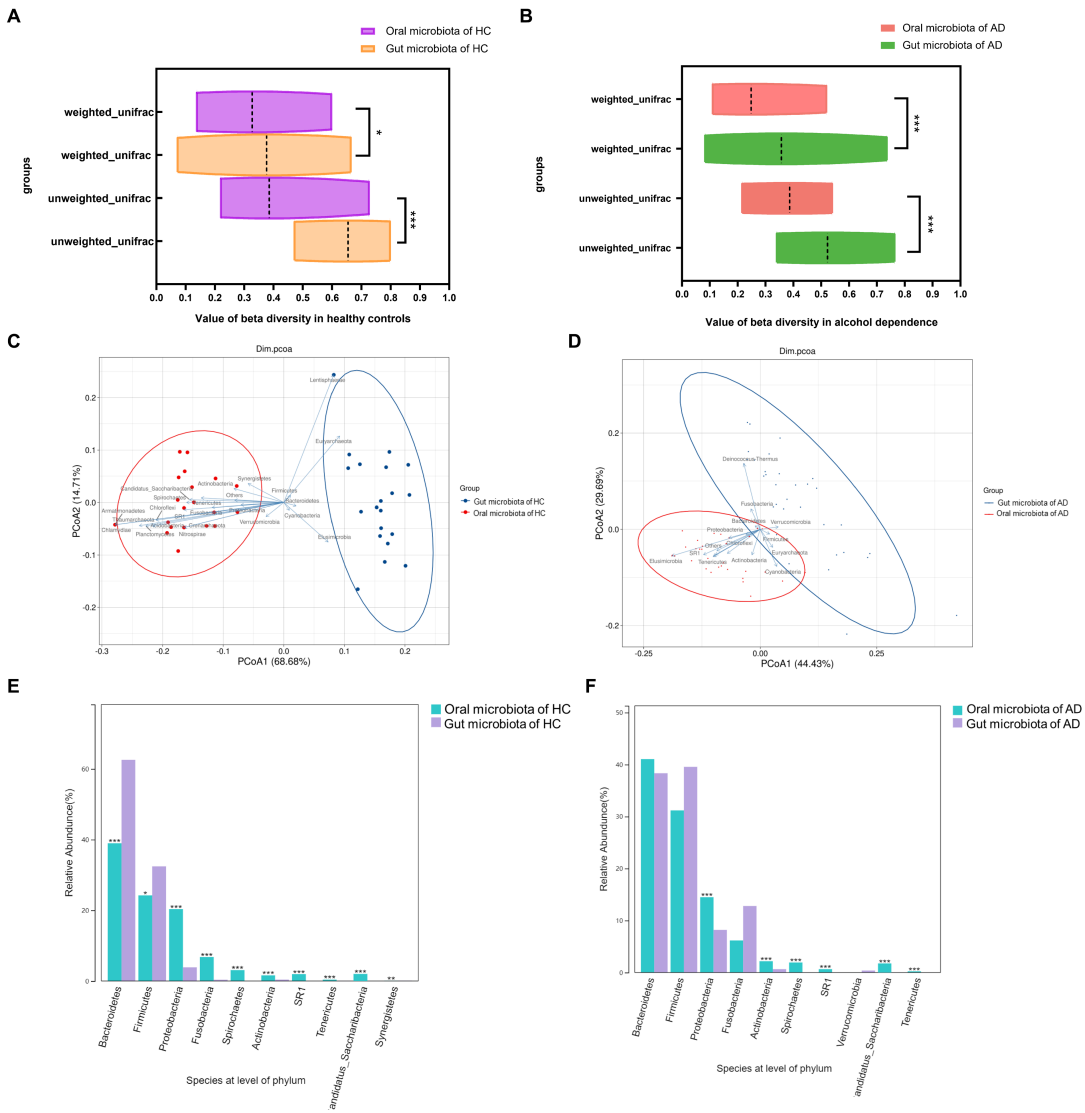
Relationships between oral and gut microbiota in alcohol dependence and healthy controls. **(A)** Difference in β-diversity between oral and gut microbiota in HC. Weight unifrac, *p* < 0.05; unweight unifrac, *p* < 0.001. **(B)** Difference in *β*-diversity between oral and gut microbiota in AD. Weight unifrac, *p* < 0.001; unweight unifrac, *p* < 0.001. **(C)** Distribution of oral and gut microbiota in HC analyzed by Principal Co-ordinates Analysis (PCoA). **(D)** Distribution of oral and gut microbiota in AD analyzed by Principal Co-ordinates Analysis (PCoA). **(E)** Significant differences between oral and gut in the top 10 species at the level of phylum in HC (all *p* < 0.05). **(F)** *Bacteroidetes*, *Firmicutes*, *Fusobacteria*, and *Verrucomicrobia* have no differences between oral and gut top 10 species at the level of phylum in AD. *Wilcoxon test indicated significant differences between different groups. All *p*-values are Bonferroni adjusted. * Indicates *p* < 0.05, ** indicates *p* < 0.01, *** indicates *p* < 0.001. AD indicates alcohol dependence, HC indicates healthy controls.

Based on unweighted Unifrac and weighted Unifrac to explore the relationship between oral and gut microbiota found that *β*-diversity between oral and gut microbiota in AD (*p* < 0.001, *p* < 0.001) and HC (*p* < 0.05, *p* < 0.001) exhibited significant difference in Figures 2A,B. Additionally, the *β*-diversity heatmap and principal coordinate analysis (PCoA) both indicate that the difference between oral and gut microbiota in AD was decreased compared to HC in Supplementary Figures S3A–C and Figures 2C,D. At the phylum level, significant differences between oral and gut microbiota were found in the top 10 species of HC (all *p* < 0.05), but four species had no significant differences between oral and gut microbiota in the top 10 species of AD in Figures 2E,F.

### The oral-gut overlap bacteria in AD

**Figure 3:**
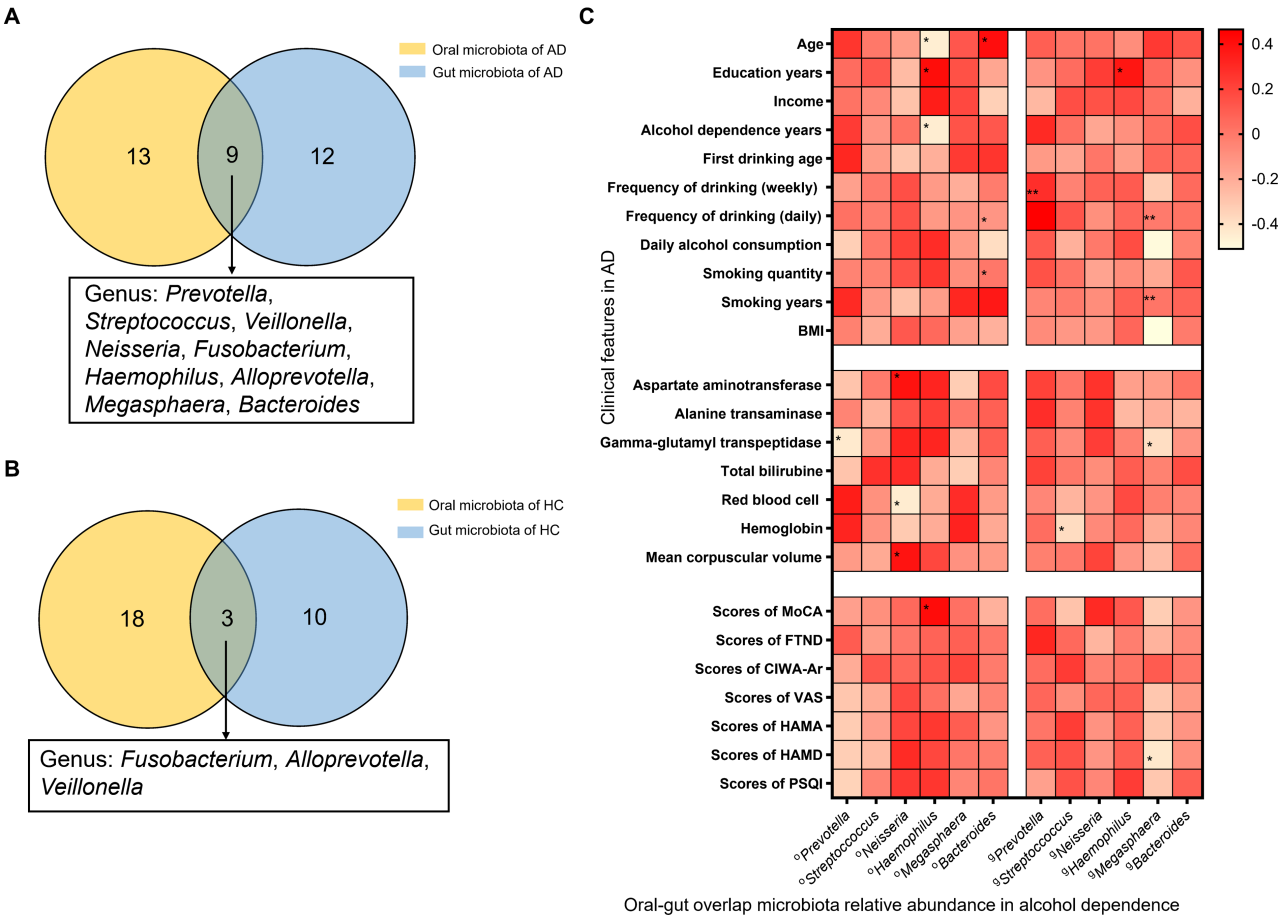
Oral and gut overlap microbiota and correlation with clinical features in AD. **(A)** There are 9 genera were oral-gut overlap bacteria, *Prevotella*, *Streptococcus*, *Veillonella*, *Neisseria*, *Fusobacterium, Haemophilus*, *Alloprevotella*, *Megasphaera*, and *Bacteroides*, in AD. **(B)** There are 3 genera were oral-gut overlap bacteria, *Fusobacterium*, *Alloprevotella*, and *Veillonella*, in HC. **(C)** Heat map correlation between oral/gut overlap bacteria and clinical features in AD. *Spearman test indicated the association between different groups. * Indicates *p* < 0.05, ** indicates *p* < 0.01. AD indicates alcohol dependence, HC indicates healthy controls.

Analysis of species composition indicated that overlaps between oral and gut microbiota were found for 9 genera in AD, including the genus of *Prevotella*, *Streptococcus*, *Veillonella*, *Neisseria*, *Fusobacterium*, *Haemophilus*, *Alloprevotella*, *Megasphaera*, and *Bacteroides*. And the genus of *Fusobacterium*, *Alloprevotella*, and *Veillonella* also are the oral/gut microbiota overlap for HC. Therefore, there were 6 AD oral/gut overlap genera, *Prevotella*, *Streptococcus*, *Neisseria*, *Haemophilus*, *Megasphaera*, and *Bacteroides*, that were not found in the HC oral/gut overlap microbiota (Figures 3A,B).

The correlation analysis between relative abundance of oral-gut microbiota and clinical features found that, in the relative abundance of oral microbiota, *Prevotella* is negatively correlated with GGT and scores of PACS (*r* = −0.43, *p* = 0.02; *r* = −0.39, *p* = 0.03); *Neisseria* is positively correlated with AST, MCV, and TSH (*r* = 0.39, *p* = 0.03; *r* = 0.39, *p* = 0.03; *r* = 0.38, *p* = 0.06), and negatively correlated with RBC (*r* = −0.44, *p* = 0.01); *Haemophilus* is positively correlated with education years, scores of MoCA (*r* = 0.42, *p* = 0.02; *r* = 0.42, *p* = 0.02), and negatively correlated with age, alcohol dependence years (*r* = −0.45, *p* = 0.01; *r* = −0.45, *p* = 0.01); *Bacteroides* is positively correlated with age, smoking years (*r* = 0.41, *p* = 0.03; *r* = 0.37, *p* = 0.04), and negatively correlated with daily alcohol consumption (*r* = −0.38, *p* = 0.04). And in the relative abundance of gut microbiota, found that *Prevotella* is positively correlated with frequency of drinking (daily) (*r* = 0.49, *p* = 0.01); *Streptococcus* is negatively correlated with HB (*r* = −0.38, *p* = 0.04) and positively correlated with TT3 (*r* = 0.39, *p* = 0.04); *Haemophilus* is positively correlated with education years (*r* = 0.38, *p* = 0.04); *Megasphaera* is negatively correlated with daily alcohol consumption, BMI, GGT, scores of PACS, scores of HAMD (*r* = −0.50, *p* = 0.01; *r* = −0.51, *p* = 0.00; *r* = −0.39, *p* = 0.03; *r* = −0.43, *p* = 0.02) (Figure 3C).

### Ectopic colonization of oral microbiota in gut based on Source Tracker

**Figure 4:**
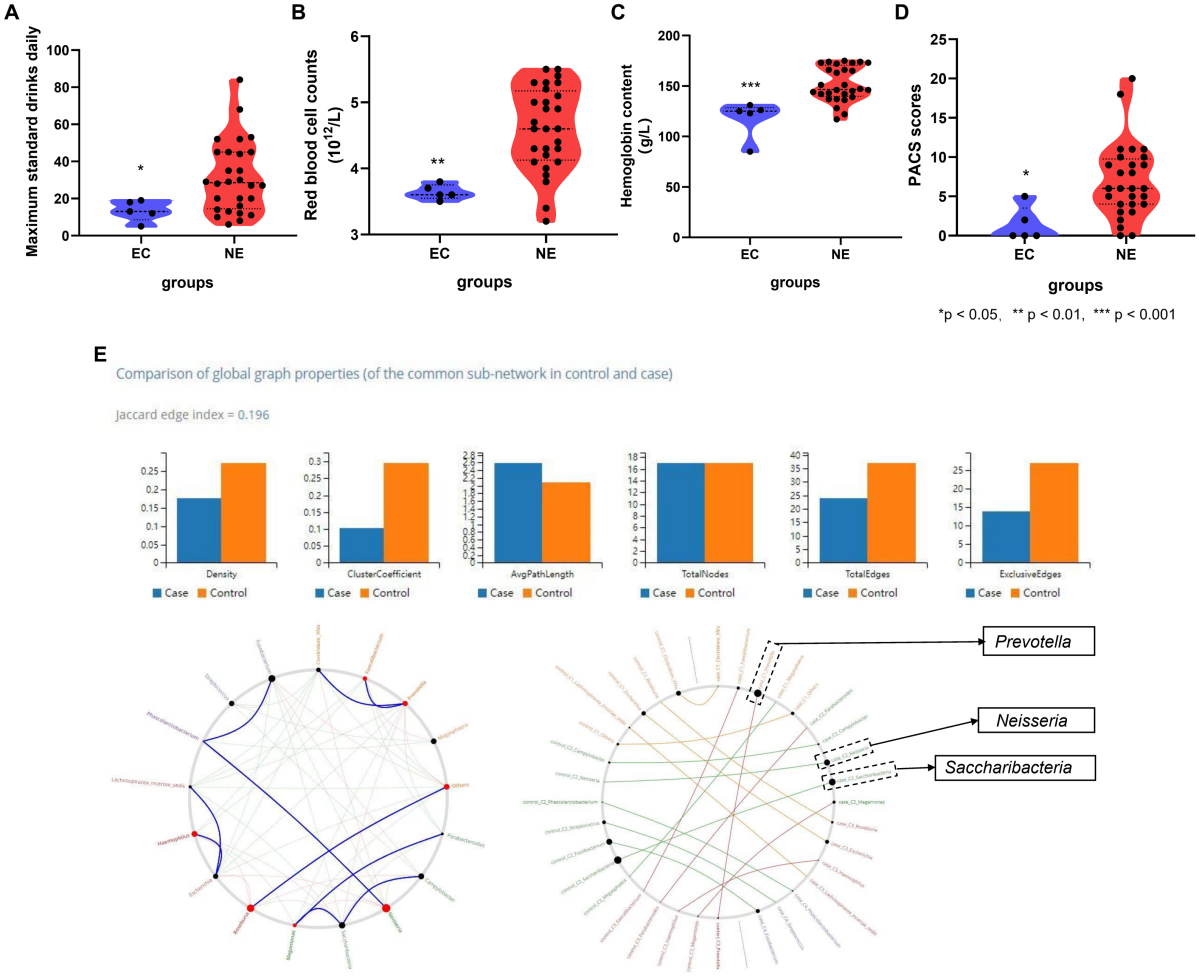
The association between clinical features and ectopic colonization bacteria in AD. **(A)** Ectopic colonization patients had lower maximum standard drinking daily (*p* < 0.05); **(B)** ectopic colonization patients had lower red blood cell counts (*p* < 0.01); **(C)** ectopic colonization patients had lower hemoglobin content (*p* < 0.001); **(D)** ectopic colonization patients had lower PACS scores (*p* < 0.05). **(E)** Genera that drive healthy states to alcohol dependence, *Prevotella*, *Neisseria*, and *Saccharibacteria*, are indicated by bigger black circles from NetShift analysis. *Mann–Whitney test indicated significant differences between different groups. *Indicates *p* < 0.05, ** indicates *p* < 0.01, *** indicates *p* < 0.001. Data are expressed as mean ± SEM (*n* = 21–33). EC indicates ectopic colonization patients, NE indicates no ectopic colonization patients, PACS indicates Pennsylvania alcohol craving scale, “Case” indicates a relative abundance of gut genera in alcohol dependence, “Control” indicates a relative abundance of gut genera in healthy controls.

Source Tracker analysis explored the ectopic colonization of oral microbiota in the gut, showing five AD, No. 3, 7, 9, 16, and 21, had a fraction of their gut microbiota which had originated from the oral cavity, but no positive results in HC. The specific oral bacteria involved in ectopic colonization, *Abiotrophia*, *Actinomyces*, *Alloprevotella*, *Bacteroidales*, *Blautia*, *and Campylobacter*…, are shown in Table 2 and Supplementary Figure S4. The contribution ratio of oral microbiota to intestinal microbiota composition in AD is 5.26%, and in HC is 0.00% based on Source Tracker. Analyze of microbiota composition between ectopic colonization and non-ectopic colonization patients, the ratio of Firmicutes to Bacteroidetes (F\B) is 1.23 for ectopic colonization and 1.06 for non-ectopic colonization compared with 0.61 for HC at the level of phylum in Supplementary Figures S5A–C. According to the Mann–Whitney U test, in contrast to AD patients without ectopic colonization, the patients with ectopic colonization showed the daily maximum standard drinks, red blood cell counts, hemoglobin content, and PACS scores decreasing (all *p* < 0.05) in Figures 4A–D. Three predominant driver bacteria, *Prevotella*, *Neisseria*, and *Saccharibacteria* in the level of the genus were found by Netshift analysis, and they were also belonging to ectopic colonization bacteria in Figure 4E and Table 2.

**Table 2:** The results of ectopic colonization based on Source Tracker (level of genus).

	Ectopic colonization (genus)
Ectopic colonization from oral microbiota in AD based on Source Tracker	*Abiotrophia*, *Actinomyces*, *Alloprevotella*, *Bacteroides*, *Blautia*, *Campylobacter*, *Capnocytophaga*, *Clostridiales*, *Corynebacterium*, *Cyanobacteria*, *Eubacterium*, *Escherichia*, *Fusobacterium*, *Flavonifractor*, *Granulicatella*, *Haemophilus*, *Leptotrichia*, *Lachnospiracea_incertae_sedis*, *Megasphaera*, *Moraxella*, *Neisseria*, *Oribacterium*, *Pasteurellaceae*, *Phocaeicola*, *Porphyromonas*, *Prevotella*, *Rothia*, *Saccharibacteria*, *Selenomonas*, *Solobacterium*, *Streptococcus*, *Treponema*, *Veillonella*
Ectopic colonization from oral microbiota in HC based on Source Tracker	Not found

### The correlation analysis between ectopic colonization bacteria and clinical features in AD

Based on the Spearman correlation test, to explore the association between ectopic colonization bacteria in the relative abundance of gut microbiota and clinical features in AD. *Alloprevotella* is positively correlated with first drinking age and ALT (*r* = 0.41, *p* = 0.03; *r* = 0.36, *p* = 0.04), *Escherichia* is negatively correlated with AST (*r* = −0.38, *p* = 0.04), *Fusobacterium* is positively correlated with MCV (*r* = −0.39, *p* = 0.03). *Lachnospiracea_incertae_sedis* is negatively correlated with frequency of drinking (weekly), AST, ALT, GGT, and TSH (*r* = −0.53, *p* = 0.00; *r* = −0.54, *p* = 0.00; *r* = −0.55, *p* = 0.00; *r* = −0.44, *p* = 0.01; *r* = −0.45, *p* = 0.0). *Megasphaera* is negatively correlated with average standard drinks per day, scores of PACS, scores of HAMD, and BMI (*r* = −0.46, *p* = 0.01; *r* = −0.42, *p* = 0.02; *r* = −0.42, *p* = 0.02; *r* = −0.52, *p* = 0.00). *Prevotella* is positively correlated with frequency of drinking (daily) (*r* = 0.47, *p* = 0.00). *Saccharibacteria* is negatively correlated with TBiL (*r* = −0.39, *p* = 0.01). *Streptococcus* is negatively correlated with HB (*r* = −0.37, *p* = 0.04) in Table 3.

**Table 3:** The correlation analysis between ectopic colonization bacteria and clinical features in alcohol dependence.

	FD age	FREW	FRED	ASD	AST	ALT	GGT	TBIL	HB	MCV	TSH	PACS	HAMD	BMI
^g^*Alloprevotella*	0.41^*^	–	–	–	–	0.36^*^	–	–	–	–	–	–	–	–
^g^*Escherichia*	–	–	–	–	−0.38^**^	–	–	–	–	–	–	–	–	–
^g^*Fusobacterium*	–	–	–	–	–	–	–	–	–	0.39^*^	–	–	–	–
^g^*Lachnospiracea_ incertae_sedis*	–	−0.53^**^	–	–	−0.54^**^	−0.55^**^	−0.44^*^	–	–	–	−0.45^*^	–	–	–
^g^*Megasphaera*	–	–	–	−0.46^*^	–	–	–	–	–	–	–	−0.42^*^	−0.42^*^	−0.52^**^
^g^*Prevotella*	–	–	0.47^**^	–	–	–	–	–	–	–	–	–	–	–
^g^*Saccharibacteria*	*–*	–	–	–	–	–	–	−0.39^*^	–	–	–	–	–	–
^g^*Streptococcus*	–	–	–	–	–	–	–	–	−0.37^*^	–	–	–	–	–

AD, alcohol dependence; FD age, first Drinking age; FREW, frequency of drinking (weekly); FRED, frequency of drinking (daily); ASD, Average Standard Drinks per day; AST, aspartate aminotransferase; ALT, alanine transaminase; GGT, gamma-glutamyl transpeptidase; TBIL, total bilirubine; HB, hemoglobin; MCV, Mean Corpuscular Volume; TSH, Thyroid Stimulating Hormone; PACS, Pennsylvania Alcohol Craving Scale; HAMD, Hamilton Depression Scale; BMI, Body Mass Index; ^g^ relative aboundence of gut microbiota; *p < 0.05，**p < 0.01.

## Discussion

The exploration of the relationship between oral and gut microbiota revealed that differences between oral and gut microbiota decreased and the oral/gut microbiota overlap increased in AD, compared with HC. Genera involved in ectopic colonization of oral microbiota to gut were shown connected with AD symptom severity based on Source Tracker and NetShift analysis. Compared with previous studies which only focused on single niche, the current study emphasized the oral-gut microbiota axis and the connection between oral and gut microbiota in AD (Liao et al., 2022; Zhao et al., 2023). To the best of our knowledge, this is the first study to explore the ectopic colonization in AD combined with methods of Source Tracker and NetShift, along with other methods, this will help to enrich the etiological hypothesis of the oral-gut microbiota axis in AD.

Heavy alcohol consumption is likely to cause disturbances of oral-gut microbiota. In current study, the *α*-diversity of oral and intestinal microbiota decreased in AD, this change may predispose patients to inflammatory responses and metabolic disorders (Karkman et al., 2017). At level the of phylum, SR1 decreased and Firmicutes and Proteobacteria increased in the oral microbiota of AD, and at the level of genus, Prevotella decreased, and Bacteroides and Fusobacterium increased in the gut microbiota of AD; such compositional change may be pro-inflammatory (Brandsma et al., 2019; Ballini et al., 2020). In summary, alcohol affects the ecological niches of both oral and gut, these could provide prerequisites for ectopic colonization of oral bacteria to the gut (Li et al., 2019).

In this study, the distances between oral and gut microbiota in AD are shortened based on PCoA, which indicated that oral and gut microbiota share a greater similarity in AD. This phenomenon also has been found in elderly people, which is believed an enhanced tendency for oral bacteria to invade the gut (Iwauchi et al., 2019). Then, the current study also found 6 oral/gut overlap bacteria, *Prevotella*, *Streptococcus*, *Neisseria*, *Haemophilus*, *Megasphaera*, and *Bacteroides* in AD. A previous study found 7 oral/gut overlap bacteria, *Actinomyces*, *Bifidobacterium*, *Dialister*, *Granulicatella*, *Lactobacillus*, *Megasphaera*, and *Veillonella* in alcohol use disorder (AUD) (Kobyliak et al., 2018; Ames et al., 2020; Maitre et al., 2020). And the difference in specific bacteria might be related to alcohol consumption, race, and dietary habits.

However, the question of what causes changes in the association between oral and gut microbiota and whether oral microbiota can ectopically colonize the gut in AD needs to be answered by the approaches of Source Tracker and NetShift. The contribution ratio of oral microbiota to the composition of intestinal microbiota in AD is 5.26%, and no ectopic colonization was found in HC. Among the current study, those showing ectopic colonization often suffered lower red blood cell counts and hemoglobin content, meanwhile, their daily maximum standard drinks and craving for alcohol were also lower than non-ectopic colonization. This reduction in craving seems to confirm the prevailing hypothesis of the work that ectopic bacteria from the oral cavity to the gut contribute to the pathological state, because low red blood cell counts and hemoglobin content represent the poor state of nutrition in AD, and impaired nutritional status often with lower levels of ghrelin and alcohol craving, and consumed high quantity of ultra-processed food had higher obsessive thoughts about alcohol (Addolorato et al., 2006; Amadieu et al., 2021). Thus, ectopic colonization in AD might be regarded as a sign of deterioration in diseases (Ley et al., 2008; Wade, 2013). Previous studies have indicated that oral microbiota is a reservoir of pathogenic bacteria (Lim et al., 2017). Colonization of the gut from oral bacteria may precipitate intestinal microbiota disturbance and barrier damage, resulting in low levels of systemic inflammation and distal spread (Bull-Otterson et al., 2013; Imai et al., 2021). In addition, HC had a proportion of the intestinal microbiota which derived from the oral microbiota of less than 1% according to the Chinese study, which similar to the results of current study (Guo et al., 2022).

The bacteria that drive state from health to AD, including *Prevotella*, *Neisseria*, and *Saccharibacteria*, were also belonging to ectopic colonization of oral bacteria based on *NetShift* analysis. Previous studies found that, *Prevotella* is an opportunistic pathogen that may increase branched-chain amino acid (BCAA) synthesis in the gut and lead to insulin resistance (Wu et al., 2011; Wang et al., 2021). *Prevotella* and *Neisseria* were also enriched in the gut of Crohn’s disease (CD) (Zhang et al., 2020). *Bacteroides* and *Prevotella* have both been shown to contribute to metabolic endotoxemia in a high-fat fed mouse model (Ding et al., 2020). *Saccharibacteria* may improve renal function through a symbiotic relationship with other host bacteria, which could decrease the concentration of serum creatinine (−0.011 [95% CI –0.019, −0.003], *p* = 0.007) and increase the estimated glomerular filtration rate (eGFR) (0.012 [95% CI 0.004, 0.020], *p* = 0.003) (Xu et al., 2020), and was found to reduce inflammation in a mouse model of periodontitis (Chipashvili et al., 2021).

**Figure 5:**
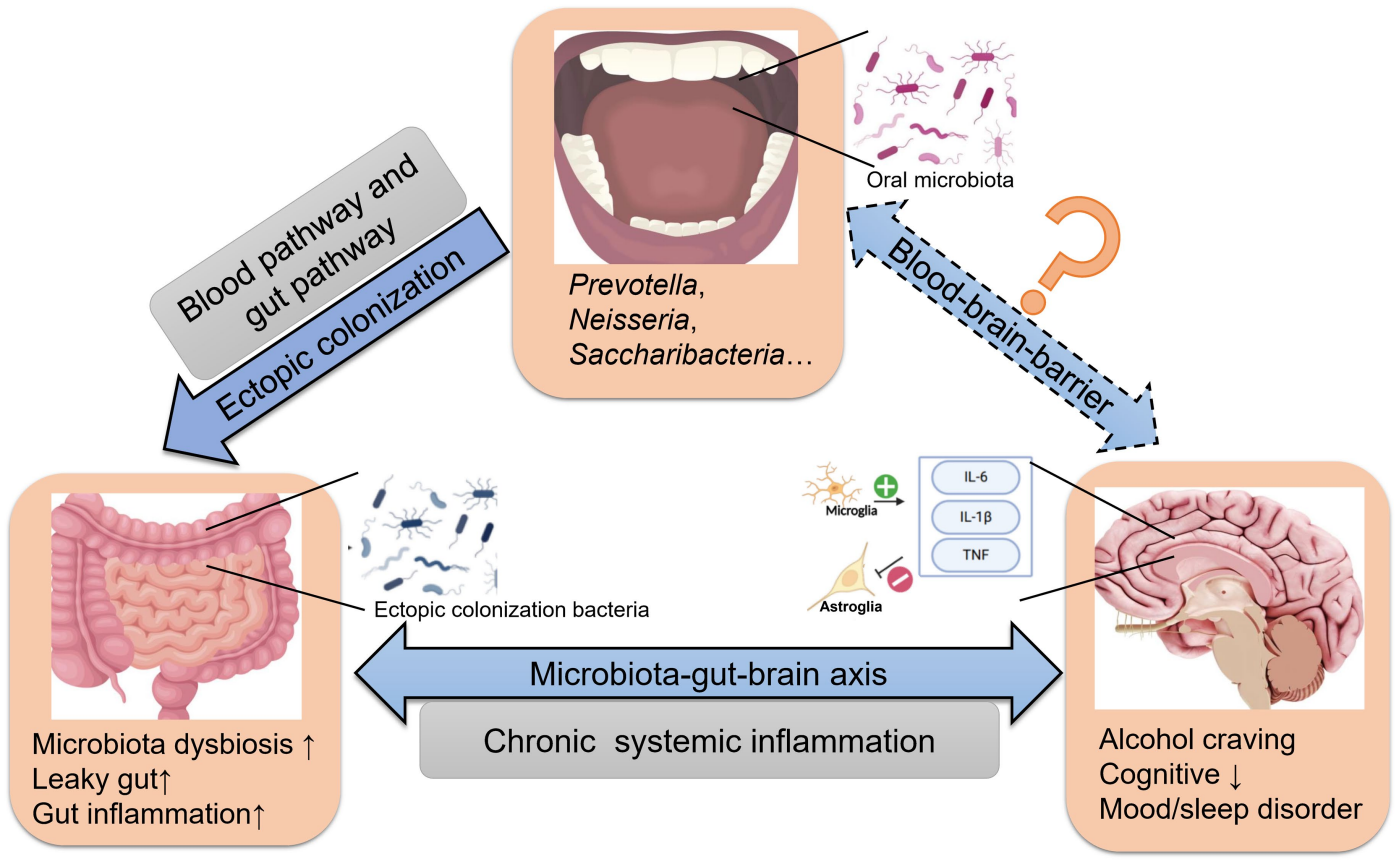
The effect of ectopically colonized bacteria on the microbiota-gut-brain axis in AD. Oral microbiota arrive at the gut *via* the blood and/or gut pathway and colonize the distal intestine. Then aggravate gastrointestinal microbiota dysbiosis, barrier injury and inflammation, ultimately affecting alcohol craving in AD.

Ectopic colonization bacteria correlated with the clinical feature in AD, such as first drinking age, frequency of drinking (weekly and daily), average standard drinks per day, BMI, AST, ALT, GGT, TBIL, HB, MCV, TSH, PACS scores and HAMD scores. Therefore, the presence of the ectopic genera may be an objective indicator of disease severity. Meanwhile, the current study also compared the species’ relative abundance of 6 oral/gut overlap bacteria with clinical characteristics, further combining the results in Figure 3C and Table 3 revealing that the ectopic colonization of the oral flora to the intestine could be one of the reasons for the increased number of oral-gut overlap bacteria in alcohol dependent, and oral ectopic colonized bacteria might play a role in the intestine and correlate with the severity of clinical symptoms. In terms of mechanism about ectopic colonization, previous research found that ectopic colonization bacteria from the oral cavity, such as *Fusobacterium nucleatum*, *Porphyromonas gingivalis*, and *Klebsiella* spp., aggravate gut dysbiosis and favor pro-inflammatory bacteria (Arimatsu et al., 2014), and activation of the NF-ĸB-PPAR-I FABP/Angptl4 pathway decreases lipid accumulation and induces intestinal apoptosis (Nakajima et al., 2015; Olsen and Yamazaki, 2019). Furthermore, *P. gingivalis* activates the MAL-PI3K pathway, and *Klebsiella spp.* resulted in potent Th1 cell differentiation in the gut (Maekawa et al., 2014; Atarashi et al., 2017; Scassellati et al., 2021). Meanwhiles, amounts of bacteria and their products invade the blood, activating B cells and inflammatory response, increasing fibrinogen production, and vascular endothelial injury in the liver (Wiest et al., 2014; Sansores-España et al., 2021). Thrombotic occlusion of capillaries and excessive interleukin-1β (IL-1β) and interleukin-6 (IL-6) secretion may follow (Leclercq et al., 2017; Imai et al., 2021). Low-grade inflammation in peripheral tissues and vagus nerve fibers (IL-1β) in the gut-brain axis may weaken the blood–brain barrier (BBB) (Maekawa et al., 2014; Acharya et al., 2017; Leclercq et al., 2017; Sansores-España et al., 2021). Inflammatory responses in the CNS may cause activated microglia to produce tumor necrosis factor (TNF), IL-1β, and IL-6 and neurotrophic factor (BDNF) from astroglia. Inflammation damages the limbic system and dorsal lateral prefrontal cortex (DLPFC) of the brain, exacerbating the cognitive impairment and affective disturbance of AD (Lee et al., 2005; Liu et al., 2016; Pascual et al., 2021) (Supplementary Figure S6). We could deduce that ectopic colonization of oral microbiota could be involved in intestinal and brain dysfunction in AD through the microbiota-gut-brain axis from the network above (Figure 5).

We acknowledge some limitations to the current study. Ectopic colonization in AD was investigated using data analysis of Source Tracker, thus it is still not enough to conclude that there is ectopic colonization in the gut by specific oral bacteria, and experimental verification is necessary. This study only included male subjects, lack of representation of females as one of the limitations of the study.

## Conclusion

The decreased divergence between oral and gut microbiota and increased oral-gut overlap bacteria were found in AD. Ectopic colonization bacteria were only found in AD based on Source Tracker. Connections between oral-gut microbiota and mechanisms of AD progression are highlighted and could be regard as the potential biomarker for oral-gut microbiota mechanisms in AD development.

## Data availability statement

The datasets presented in this study can be found in online repositories. The names of the repository/repositories and accession number(s) can be found at: https://www.ncbi.nlm.nih.gov/, PRJNA895096; https://www.ncbi.nlm.nih.gov/, PRINA905862.

## Ethics statement

The studies involving human participants were reviewed and approved by the Helsinki Declaration of 1975 and the ethical standards of the home institution Committee on Human Experimentation. Ethical approval was granted by the ethics committees of Peking University Sixth Hospital (2019, ethical review No. 6) and the Second People’s Hospital of Guizhou Province. The patients/participants provided their written informed consent to participate in this study.

## Author contributions

LH, ZN, LL, and HS were responsible for the study concept and design. KZ contributed to the acquisition of human data. LH, YK, and ZY performed the gut and oral microbiota analysis. YK and XG assisted with data analysis and interpretation of findings. XL provided critical revision of the manuscript for important intellectual content. All authors critically reviewed the content and approved the final version for publication.

## Funding

Our study got support from the National Natural Science Foundation of China (nos. 81971235, 81771429, and 81571297) and the National Clinical Research Center for Mental Disorders (Peking University Sixth Hospital) NCRC2020M09.

## Conflict of interest

The authors declare that the research was conducted in the absence of any commercial or financial relationships that could be construed as a potential conflict of interest.

## Publisher’s note

All claims expressed in this article are solely those of the authors and do not necessarily represent those of their affiliated organizations, or those of the publisher, the editors and the reviewers. Any product that may be evaluated in this article, or claim that may be made by its manufacturer, is not guaranteed or endorsed by the publisher.

## Abbreviations

AD: alcohol dependence
BMI: body mass index
CIWA-Ar: Revised Clinic Institute Alcohol Withdrawal Syndrome Assessment Scale
DSM-IV: 4th edition of the Diagnostic and Statistical Manual of Mental Disorders
F/B: The Ratio of Firmicutes to Bacteroidetes
FTND: Fagerstrom Test of Nicotine Dependence
HAMA: Hamilton Anxiety Scale
HAMD: Hamilton Depression scale
M.I.N.I: Mini-International Neuropsychiatric Interview
MoCA: Montreal cognitive assessment
OTUs: operational taxonomic units
PACS: Pennsylvania Alcohol Craving Scale
PCoA: Principal Co-ordinates Analysis
PSQI: Pittsburg Sleep Quality Index
